# Polydatin ameliorates lipid and glucose metabolism in type 2 diabetes mellitus by downregulating proprotein convertase subtilisin/kexin type 9 (PCSK9)

**DOI:** 10.1186/s12933-015-0325-x

**Published:** 2016-02-01

**Authors:** Yu Wang, Jiantao Ye, Jie Li, Cheng Chen, Junying Huang, Peiqing Liu, Heqing Huang

**Affiliations:** Laboratory of Pharmacology and Toxicology, School of Pharmaceutical Sciences, Sun Yat-sen University, 132 WaiHuan East Road, Guangzhou Higher Education Mega Center, Guangzhou, 510006 China; Laboratory Animal Center, Sun Yat-sen University, Guangzhou, 510080 China; National and Local United Engineering Lab of Druggability and New Drugs Evaluation, Guangzhou, 510006 China

**Keywords:** Dislipidemia, Hyperglycemia, Insulin resistance, PCSK9, LDLR, GCK

## Abstract

**Background:**

Abnormalities in lipid and glucose metabolism are constantly observed in type 2 diabetes. However, these abnormalities can be ameliorated by polydatin. Considering the important role of proprotein convertase subtilisin/kexin type 9 (PCSK9) in metabolic diseases, we explore the possible mechanism of polydatin on lipid and glucose metabolism through its effects on PCSK9.

**Methods:**

An insulin-resistant HepG2 cell model induced by palmitic acid (PA) and a db/db mice model were used to clarify the role of polydatin on lipid and glucose metabolism.

**Results:**

In insulin-resistant HepG2 cells, polydatin upregulated the protein levels of LDLR and GCK but repressed PCSK9 protein expression, besides, polydatin also inhibited the combination between PCSK9 and LDLR. Knockdown and overexpression experiments indicated that polydatin regulated LDLR and GCK expressions through PCSK9. In the db/db mice model, we found that polydatin markedly enhanced GCK and LDLR protein levels, and inhibited PCSK9 expression in the liver. Molecular docking assay was further performed to analyze the possible binding mode between polydatin and the PCSK9 crystal structure (PDB code: 2p4e), which indicated that steady hydrogen bonds formed between polydatin and PCSK9.

**Conclusions:**

Our study indicates that polydatin ameliorates lipid and glucose metabolism in type 2 diabetes mellitus by downregulating PCSK9.

## Background

Lipid and glucose metabolism disorders are the main characteristics of insulin-resistant type 2 diabetes mellitus [1]; these disorders are also the basic pathology in diabetic microvascular complications [2]. Stable glucose-lowering and lipid-lowering therapies effectively retard the progression of chronic diabetic microvascular complications [3, 4]. Therefore, exploring the agents with both hypoglycemic and hypolipidemic effects to treat diabetes and its complications is necessary. Drugs that regulate metabolic diseases by targeting PCSK9 have recently drawn great attention all over the world [5–9]. Several studies have demonstrated that PCSK9 plays a vital role in the degradation of low-density lipoprotein receptor (LDLR) [10–13]. PCSK9 binds to LDLR and then, reroutes it from the endosome to the lysosome, where the LDLR is degraded rather than recycling back to the cell membrane, thereby leading to an impaired cholesterol uptake and elevated serum cholesterol levels [10–13]. Both the animal experiments and clinical studies have suggested that PCSK9 is also closely linked to glucose metabolism [14–18] and triglyceride levels [16, 19]. Repressing PCSK9 expression can control serum lipids and thus, ameliorate insulin resistance to some extent. As such, PCSK9 might be a promising target in ameliorating lipid and glucose metabolism disorders and improving insulin resistance.

Polydatin (resveratrol-3-O-β-mono-d-glucoside), also known as piceid, is a major active component of *Polygonum cuspidatum* Sieb. et Zucc. It is a glycoside of resveratrol. Previous studies have shown that polydatin exerts several pharmacological effects, including anti-inflammation [20–23], anti-oxidant [24, 25], anti-allergy [26], anti-cancer [27], lipid-lowering [28, 29], and cardiovascular- protection effects [30, 31]. We found that polydatin could improve lipid and glucose metabolism in STZ-induced diabetic rats and regulate GCK and LDLR expression [32]. Considering the close relationship between PCSK9 and LDLR, as well as insulin resistance, we sought to determine whether polydatin works by affecting PCSK9.

Based on the above background, we chose an insulin-resistant HepG2 cell model induced by PA [32] and a db/db mice model to explore the exact effects of polydatin on PCSK9, LDLR, GCK, and other metabolic parameters. To further elucidate its interaction with PCSK9, polydatin was docked into the active pocket of the PCSK9 crystal structure using Surflex-Dock in Sybyl 7.3.5 to analyze the specific binding motifs between polydatin and PCSK9. Our results demonstrate that polydatin ameliorates lipid and glucose metabolism in type 2 diabetes mellitus by downregulating proprotein convertase subtilisin/kexin type 9 (PCSK9).

## Methods

### MTT cell proliferation assay

The 3-(4, 5-dimethylthiazol-2-yl)-2, 5-diphenyl tetrazolium bro-mide (MTT, Sigma, USA) assay was used to detect cell viability of HepG2 cells for increasing concentrations of polydatin under insulin resistant condition induced by PA. Briefly, cells were seeded in 96-well plate, and incubated with 0.25 μM PA for 24 h with or without polydatin after cell subconfluence. Then 20 μl of MTT (0.5 mg/ml) was added to each well and incubation continued at 37 °C for an additional 4 h. The medium was then carefully removed, so as not to disturb the formazan crystals formed. Dimethyl sulphoxide (DMSO, 200 μl, Sigma, USA), which solubilizes the formazan crystals, was added to each well and the absorbance of solubilized blue formazan was read at wave-length of 570 nm using a microplate reader (Bio-Tek, USA). The reduction in optical density caused by polydatin used as a measurement of cell proliferation, normalized to cells incubated in control medium, which were considered 100 % viable.

### Insulin resistant cell model and the treatment of polydatin

HepG2 cells (American Type Culture Collection, Rockville, MD,USA) were grown at 37 °C in high-glucose DMEM (Gbico, Invitrogen, USA) containing: 10 % (v/v) FBS (Gibco, Invitrogen, USA), 100 U mL^−1^ penicillin, 100 mg mL^−1^ streptomycin (Hyclone, USA) and 1 % l-glutamine (Sigma, USA). Cells were grown in a humidified atmosphere of 95 % air/5 % CO_2_ at 37 °C, and in six multi-well plates at proper cell densities. At appropriate subconfluence, the HepG2 cells were serum-starved for 12 h and then divided into different groups for different treatments. Cells were preincubated with the presence or absence of polydatin (Chuangwei, Beijing, China) at the dosage of 5, 10, 20, and 40 μM for 1 h, and then stimulated with or without 0.25 mM of PA (Sigma, USA), which was prepared as previous study [33] for another 24 h. Considering that PA was dissolved in BSA (low free fatty acid, MP Biomedical, USA), 0.5 % BSA was added as normal control. All experiments were performed in triplicate.

### Animal model

Twenty-one healthy specific pathogen free female db/db leptin receptor deficient type 2 diabetic mice (abbreviated by db/db) aged 6 weeks and seven female wild type C57BL/6 mice (abbreviated by C57) aged 6 weeks were supplied by the Experimental Animal Center of Sun Yat-sen University (Guangzhou, China; animal quality certification number: 201403212). The mice were adapted to the environment for 1 week and then randomly divided into 4 groups based on the weight and fasting blood glucose (FBG) levels as follows: C57 control group (n = 7), db/db model group (n = 7), polydatin treatment group (n = 7, 100 mg/kg, dissolved in 0.5 % (w/v) CMC-Na; purity >98 %, HPLC; Zelang, Nanjing, China), and pioglitazone treatment group (n = 7, 10 mg/kg, dissolved in 0.5 % (w/v) CMC-Na; Takeda, Japan, subpackaged by Tianjin Takeda). The mice were administered by gavage 6 days every week at 9:30–10:30 am, totally for 4 weeks. We weighed the mice every day to determine the exact dose of drugs needed to be given and measured the FBG levels every other week using a One-Touch glucometer (Johnson and Johnson, USA) after starvation for 12 h. The animals were housed in a temperature-controlled (20–25 °C) and humidity-controlled (40–70 %) barrier system with 12:12 h light and dark cycle.

### Plasma and liver tissue collection

At the end of the experiment, all animals were fasted for 12 h and blood sample was collected by drainage from the retroorbital venous plexus after the FBG detection. Serum was obtained by centrifuge at 3000*g* for 15 min and stored at −80 °C to detect the metabolic parameters. Liver samples were quickly weighed and excised into same parts, and a small part was fixed in 10 % neutral buffered formalin and subsequently embedded in paraffin or for further Oil Red O staining, and the left was frozen in liquid nitrogen immediately and stored at −80 °C for other assays.

### Lipid profile and PCSK9 level in serum

The serum was balanced at room temperature for 30 min and diluted with NaCl solution (0.9 %) by 1:1. Then the total cholesterol (TC), triglyceride (TG), lower density lipoprotein cholesterol (LDL-C), and high density lipoprotein cholesterol (HDL-C) levels were detected using the auto-analysis biochemical instrument (Beckman coulte CX5). Serum PCSK9 level was detected following the instruction of the Human PCSK9 ELISA Kit (CUSABIO; Wuhan, China).

### The content of TC, TG, and glycogen in liver

The frozen liver tissues were rinsed, dried, and weighed for hepatic TC, TG, and glycogen measurement according to the manufacturer’s instructions (Jiancheng; Nanjing, China). Briefly, 100 mg liver samples were added to 0.2 ml 0.9 % NaCl buffer and homogenized by using a homogenizer on ice, followed by the extraction step with a solvent having a 2:1 (v/v) chloroform-to- methanol ratio at room temperature. After mingled thoroughly, the samples were stewing for 18 h at room temperature and then the samples were separated into three parts: water phase (the upper layer), tissue fragment (the middle layer), lipid phase (the lowest layer). The lipid phase was collected carefully to detect the TC and TG levels in liver. The glycogen was hydrolyzed in base solutions for 20 min in boiling water and then diluted the sample to 1 % solution to detect the glycogen content.

### H&E staining

Ten percent neutral formalin-fixed paraffin-embedded liver sections (3–4 μm) were stained with hematoxylin and eosin (H&E) using standard protocols. Briefly, the sections were deparaffinization, rehydrated, stained with hematoxylin and agitation for 30 s, stained the slide with 1 % eosin solution for 20 s after being washed in H_2_O, dehydrated the sections and covered with neutral balsam.

### Oil Red O staining

The livers were fixed for over 24 h, dehydrated in 15 and 30 % sucrose solutions at −4 °C in succession, OCT- embedded, cold-sliced (8–10 μm), and refixed at room temperature for another 15 min, Oil Red O stained for 15 min, differentiated with 75 % alcohol, counterstained with hematoxylin for 2 min, and finally covered with neutral balsam.

### Western blot assay

Firstly, the lysis buffer was prepared by adding protease and phosphatase inhibitor cocktail (100×; Thermo, USA) to RIPA lysis buffer (pH 8.0, 50 mM Tris, 150 mM NaCl, 0.02 % sodium azide, 0.1 % SDS, 1 % NP-40, 0.5 % sodium deoxycholate, 1 mM EDTA, and so on). For the cells, the plate was rinsed with ice-cold PBS buffer for three times and then collected the cells with the lysates. For the liver tissues, samples were added to cold lysis buffer and homogenized by using a homogenizer. The lysates or homogenates were centrifuged and then, the supernatant was collected. Equal amounts of protein samples were separated by 8 % (v/v) SDS-PAGE and electrophoretically transferred onto a nitrocellulose membrane. After blocking with 5 % nonfat milk at room temperature for 1.5 h, the membranes were incubated with the corresponding primary anti-LDLR (1:1000; Santa Cruz, CA, USA), anti-GCK (1:1000; Santa Cruz, CA, USA), anti-PCSK9 (1:500; Santa Cruz, CA, USA), and anti-Tubulin (1:10,000; Sigma, USA) antibodies at 4 °C overnight. The membranes were then incubated with horse radish peroxidase-conjugated secondary antibodies (anti-rabbit IgG or anti-mouse IgG, 1:10,000; Promega, USA) for 1.5 h at room temperature, and the immunoreactive protein bands were visualized using enhanced chemiluminescence reagents (Thermo Fisher Scientific; Rockford, IL, USA) with a GE ImageQuant LAS 4000 mini (GE healthcare; Waukesha, WI, USA). The intensity of protein bands was quantitated using a Image J analysis software (Version 1.46r, Scion, Frederick; MD, USA).

### Quantitative real-time PCR

Quantitative real-time PCR, which was performed with some modifications as described previously [34], was used to determine the relative mRNA expression levels. Briefly, total RNAs were prepared using RNAiso plus (Takara, Japan). The cDNA from the total RNA was synthesized with a PrimeScript™ RT Reagent Kit (Takara, Japan). PCR amplification for each gene was performed using SYBR^®^ Green (Toyobo, Japan) according to the manufacturer’s protocol, and the results were normalized to β-actin expression. The primers were synthesized by Invitrogen (Shanghai, China) as described in previous study [35]. Mouse LDLR primers (Forward: ACCTGCCGACCTGATGAATTC, Reverse: GCAGTCATGTTCACGGTCACA); Mouse PCSK9 primers (Forward: TTGCAGCAGCTGGGAACTT, Reverse: CCGACTGTGATGACCTCTGGA). Mouse β-actin primers (Forward: TGCGTGACATCAAAGAGAAG, Reverse: GATGCCACAGGATTCCATA).

### Immunohistochemistry and double immunofluorescence staining

Sections of liver (4 μm thick) were processed using a standard immunostaining protocol. Briefly, after deparaffinization, hydration and blockage of endogenous peroxidase routinely, sections were pretreated by microwave for 20 min in EDTA recovery buffer for antigen retrieval, followed by incubation sequentially with blocking agent, rabbit polycolonia PCSK9 antibody (1:100; Santa Cruz, CA, USA) and mouse monocolonia LDLR antibody (1:100; Santa Cruz, CA, USA) and corresponding secondary antibody (1:200; KPL, USA). Slides were counterstained with hematoxylin (Google Biotech, Wuhan, China) after 3 min of diam-inobenzidine (DAKO, CA, USA) reaction, and covered slices using neutral balsam (Sinopharm Chemical Reagent Co., Ltd, Shanghai, China), then photographed and converted to a digital image using light microscopy equipped with camera (NIKON DS-U3/LSM700; CARL ZEISS). Negative control was carried out by omitting the primary antibody and revealed no labeling (data not shown). The double immunofluorescence staining was similar with the above steps except that both the LDLR and PCSK9 primary antibody (1:100/1:100) were co-incubated in the same slide, followed by fluorenscence-conjuated secondary antibodies (1:400/1:300, respectively) and DAPI (Google Biotech, Wuhan, China) staining in nuclear in dark.

### Co-immunoprecipitation (Co-IP)

20 μl of protein agarose A/G beads (calbiochem^R^, USA) was added into 300 μg of protein lysates for pre-clearance of non-specific interaction. The supernatant was mixed in 1 μg of PCSK9 antibody (5 μl) and incubated at 4 °C overnight. The next day, 20 μl of protein agarose A/G beads was added and incubated at 4 °C for another 2 h. After centrifuging at 4 °C, 12,000*g* for 30 s, the supernatant was carefully divided without touching the beads. Rinsed the beads with indicated washing buffer for 3 times and mingled with the loading buffer finally to perform the western blot and proteins were pulled down using their specific antibodies. Rabbit IgG (Beyotime Biotech, Shanhai, China).

### PCSK9 small interfering RNA (siRNA) assay

Three pairs of specific siRNA targeting PCSK9 were designed and synthesized by GenePharma (Shanghai, China). Through western blot assay, we screened out the siRNA numbered 1153 with the best interfering effect. The sequences of siRNA-1153 used in the sequent experiment were as following: sence: 5′-CCCUCAUAGGCCU GGAGUUTT-3′; antisence: 5′-AACUCCAGGCCUAUGAGGGTT-3′. 5 μl of siRNA-1153 and 5 μl of RNAiMAX (Invitrogen, Shanhai, China) were diluted in 150 μl of opti-MEM (Invitrogen, Shanhai, China), respectively, and then mingled together for a 5-min incubation. Thereafter the mixed reagent was added into the HepG2 cells for 24 h.

### PCSK9 wild type plasmid overexpression

HepG2 cells were plated in six-well dishes and were infected with empty vector (pENTER) and PCSK9 wild type plasmid (Vigene, Shandong, China), respectively, at a multiplicity of Lipofectamine^®^ LTX and Plus (Invitrogen, Shanhai, China) in opti-MEM (Invitrogen, Shanhai, China) for 24 h.

### Molecular docking

Surflex-Dock in Sybyl 7.3.5 (Tripos, Inc., St. Louis, MO, USA) that was applied to study molecular docking uses a potent search engine to dock ligands into a protein’s binding site [36]. Polydatin was docked into the active pocket of PCSK9 crystal structure using Surflex-Dock to analyze the specific motifs between polydatin and PCSK9. The PCSK9 crystal structure was obtained from PDB (PDB code: 2p4e).

## Results

### Polydatin up-regulated the protein expressions of LDLR and GCK but down-regulated PCSK9 level in PA-induced insulin-resistant HepG2 cells

**Fig. 1:**
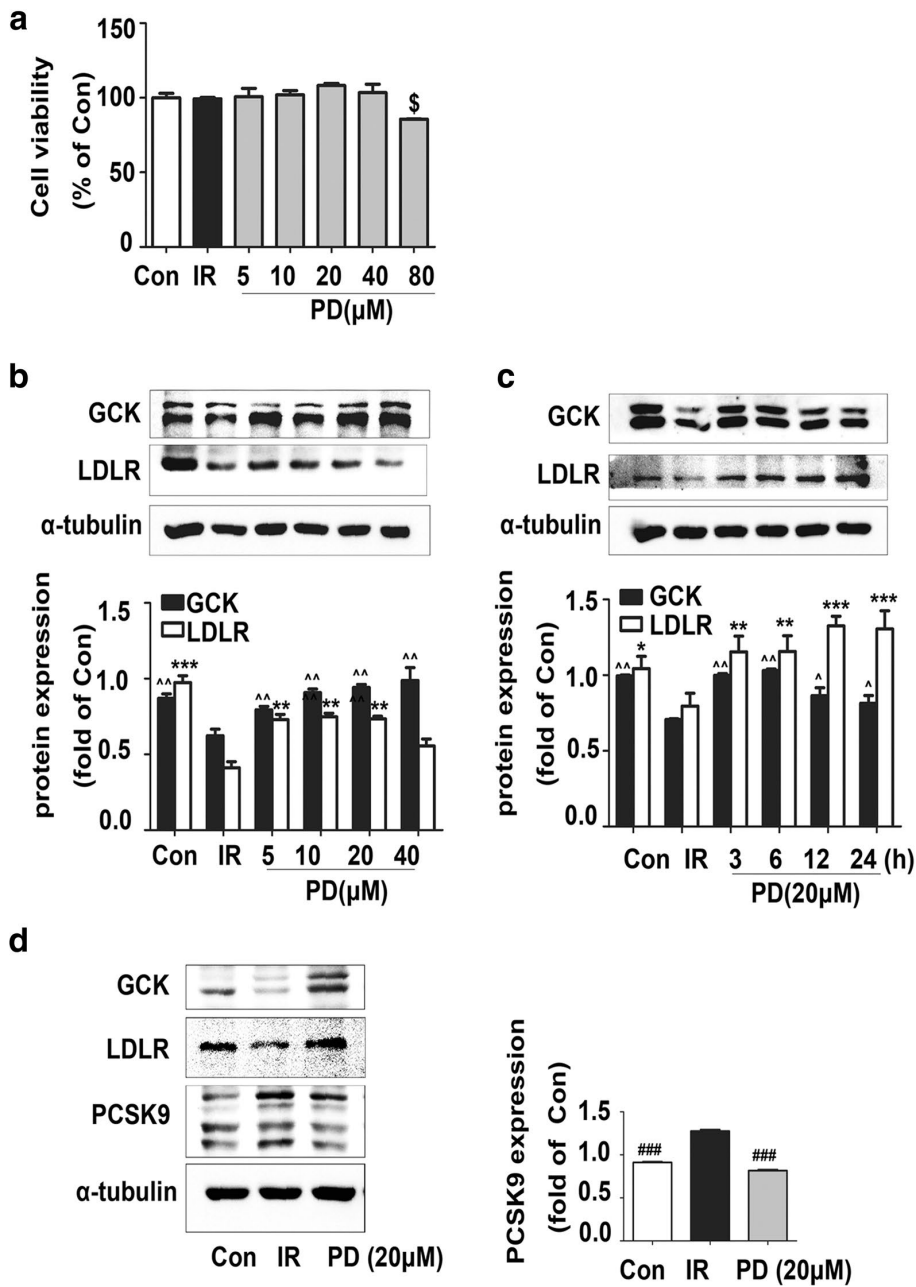
In PA-induced insulin resistance model, the expression of PCSK9, LDLR, and GCK after polydatin treatment. **a** Insulin resistant HepG2 cells were incubated with polydatin of the indicated concentrations for 24 h and measured by MTT assay. **b** LDLR and GCK protein expression with polydation treatments in 5, 10, 20, 40 μM for 24 h. **c** LDLR and GCK protein expression with polydation treatments in 20 μM for different time. **d** PCSK9, LDLR, and GCK expression with polydatin treatment in 20 μM for 24 h. All experiments were performed with polydatin (indicated concentration) pretreatment for 2 h, and then incubated with 0.25 mM of PA for another 24 h to induce insulin resistance for more than three times. Intensities were quantified and normalized against the level of α-tubulin abundance in corresponding BSA-treated cells. ^$^ *p* < 0.05 vs. Con; *p < 0.05, **p < 0.01, and ***p < 0.001 vs. LDLR levels in the control group (Con); ^^^ *p* < 0.05 and ^^^^ *p* < 0.01 vs. GCK levels in the control group (Con); ^*###*^ *p* < 0.001 vs. IR group

In insulin-resistant HepG2 cells, polydatin showed no cytotoxicity below 80 μM for 24 h in the MTT assay (Fig. 1a), the protein expressions of LDLR and GCK (Fig. 1b, c) were downregulated, which were obvious at the concentration of 20 μM for 24 h (*P* < 0.01). We then detected the protein expression of PCSK9, a protein that binds to LDLR and induces its degradation, and found that PCSK9 level was increased under the insulin-resistant conditions, which could be reversed by polydatin treatment (Fig. 1d) as expected (*P* < 0.001). Therefore an assumption that polydatin regulates LDLR and GCK levels through down-regulating PCSK9 arouses our interest.

### Polydatin regulated LDLR and GCK through PCSK9

**Fig. 2:**
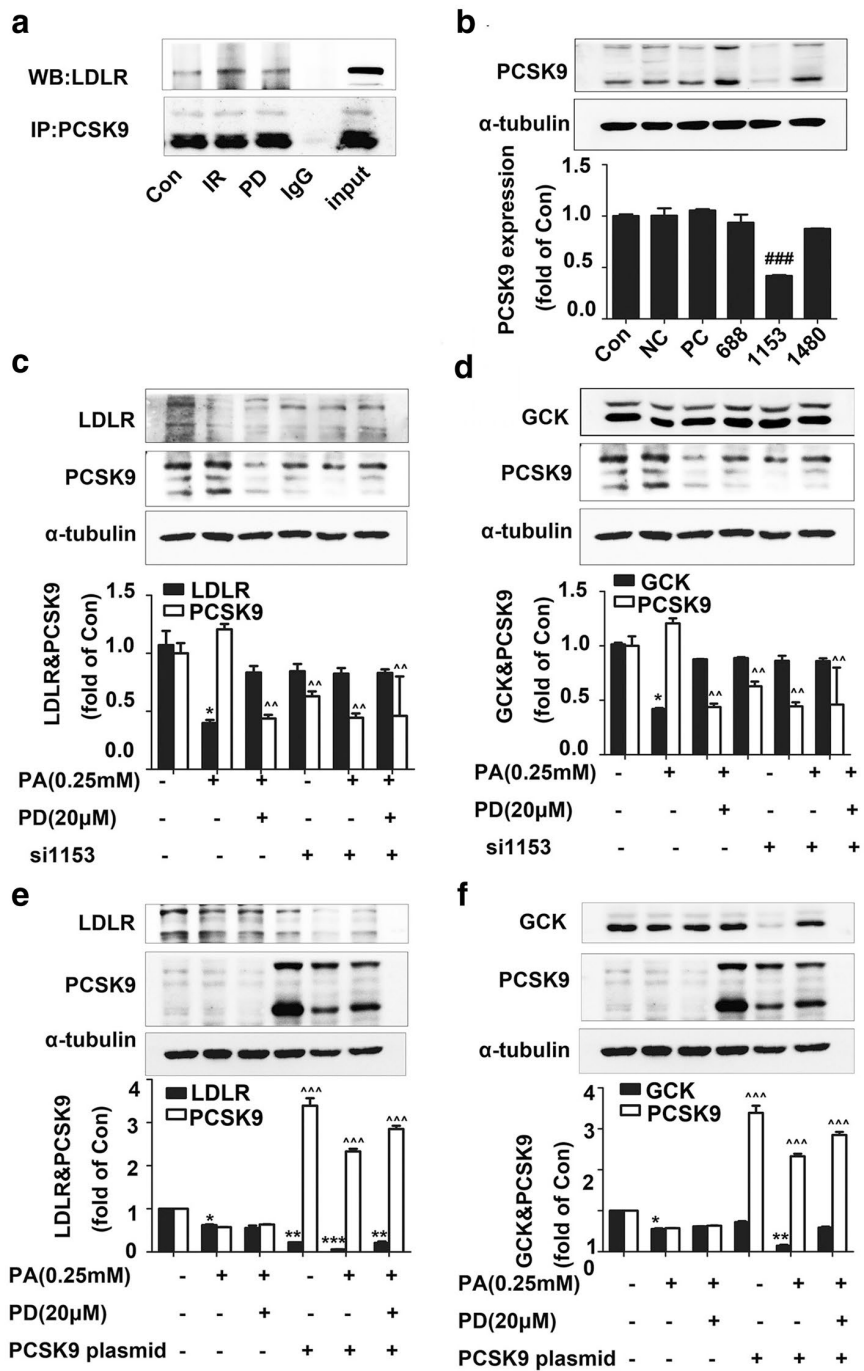
Polydatin regulated LDLR and GCK expression possibly through PCSK9. **a** Co-IP assay was performed to explore whether polydatin affected the combination of PCSK9 and LDLR according to the “Methods” above. **b** The interfering efficiency of PCSK9 by siRNA was screened, and the second sequence si1153 was chosen for subsequent experiments and the negative sequences were used as control. **c**, **d** LDLR and GCK expression under PCSK9 knockdown conditions. **e**, **f** LDLR and GCK expression under PCSK9 overexpression conditions. Overexpression of PCSK9 with Human wild-type PCSK9 plasmids and the vector PENTER plasmid were used as control. SiRNA and overexpression experiments were all divided into three groups: Control group, IR group, and IR + polydatin group. The first three lanes were treated with the negative SiRNA squences or pENTER vector as control and the last three lanes were treated with siRNA-1153 sequences or Human wild-type PCSK9 plasmids, respectively. ^*###*^ *p* < 0.001 vs. control group (Con); *p < 0.05, **p < 0.01 and ***p < 0.001 vs. LDLR levels in the control group (Con); ^^^^ *p* < 0.01 and ^^^^^ *p* < 0.001 vs. PCSK9 levels in the control group (Con)

As mentioned above that PCSK9 binds to LDLR, the Co-IP assay showed that PCSK9 interacted with LDLR in control cells, which was augmented in the insulin-resistant HepG2 cells (Fig. 2a). Polydatin treatment obviously inhibited the combination between PCSK9 and LDLR (Fig. 2a). To further explore the possible effects of PCSK9 on LDLR and GCK, we carried out a series of experiments using PCSK9 siRNA and overexpressing plasmids.

We first detected the silencing efficiency of PCSK9-siRNA. The protein levels of PCSK9 were efficiently depleted by 60 % after PCSK9-siRNA 1153 treatment as confirmed by western blot assay (Fig. 2b). Concomitant with decreased PCSK9 levels, siRNA-1153 could increase the expression of LDLR under the PA treatment conditions compared with the control group (Fig. 2c). However, the GCK level was not affected by siRNA-1153 (Fig. 2d). Interestingly, after PCSK9 was interfered by siRNA, the upregulated effects of LDLR and GCK induced by polydatin under insulin-resistant conditions disappeared (Fig. 2c, d).

Overexpression of the PCSK9 wild-type plasmid caused a sharp decline in LDLR and GCK expressions in the insulin-resistant group; these decreases, however, were restored by polydatin to nearly control levels (Fig. 2e, f).

Accordingly, polydatin upregulated the protein expressions of LDLR and GCK possibly through the inhibition of PCSK9 in PA induced insulin resistant HepG2 cells.

### Polydatin ameliorated lipid and glucose disorders and protected the liver in db/db mice

**Fig. 3:**
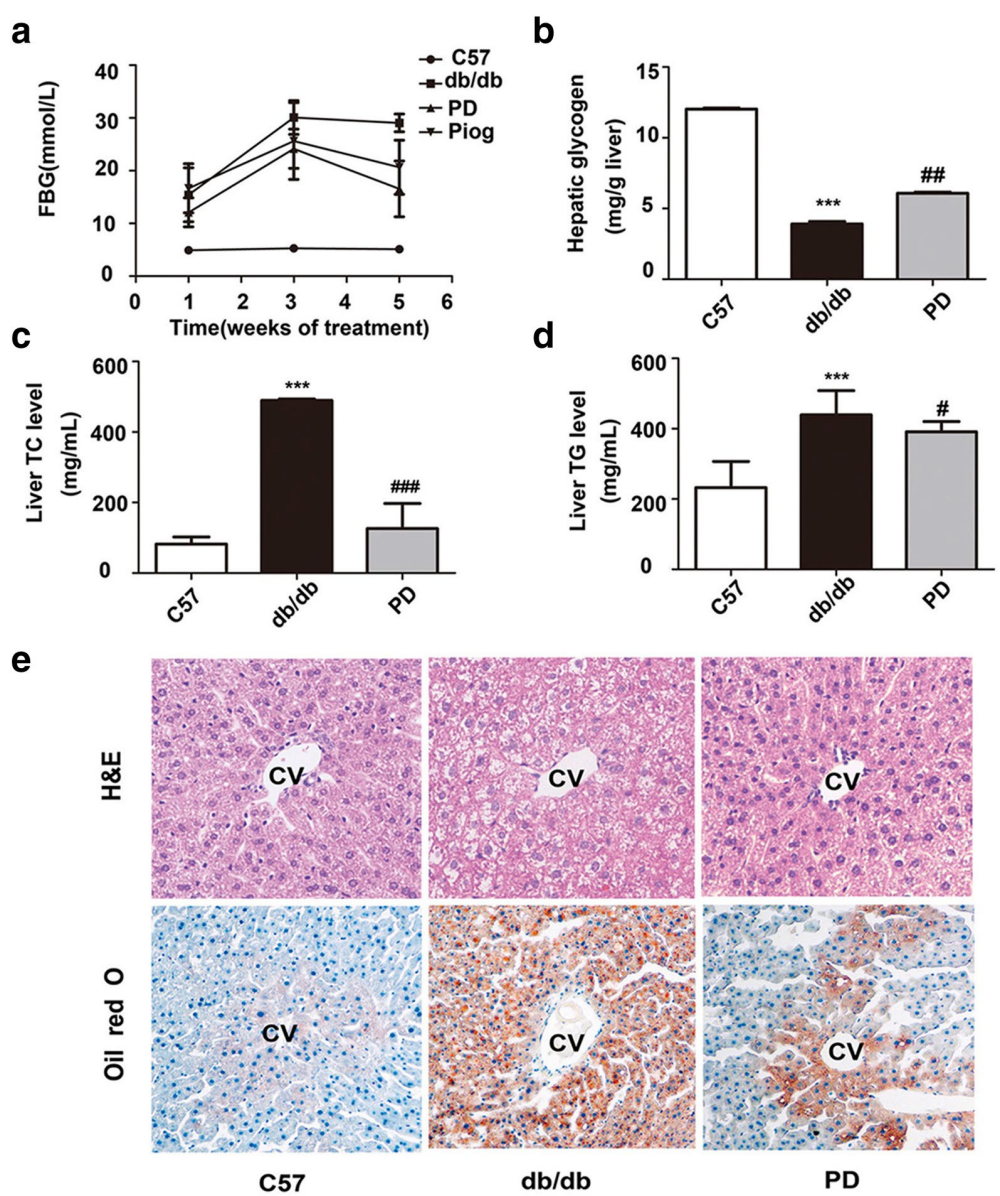
Polydatin ameliorated lipid and glucose metabolism and protected the liver from steatosis in db/db mice. **a** Fasting blood glucose levels (FBG) were measured every other week. **b**–**d** In the liver (n = 6), glycogen, TC and TG levels were determinated as described in “Methods”. **e** Histochemical analysis of fresh liver tissue was performed by staining with H&E or Oil Red O (×400 magnification). ***p < 0.001 vs. C57; ^*#*^ *p* < 0.05, ^*##*^ *p* < 0.01 and ^*###*^ *p* < 0.001 vs. db/db, *cv* center vain

In order to confirm the effects of polydatin on lipid and glucose metabolism, we testified it in the db/db mice model. Polydatin attenuated FBG whose changes observed were slightly better than that in the pigolitazone treatment group (Fig. 3a) and decreased total cholesterol (TC), triglyceride (TG), and low-density lipoprotein cholesterol (LDL-C) levels (Table 1). Furthermore, polydatin significantly increased glycogen contents (Fig. 3b) and decreased TC and TG accumulation in the liver (Fig. 3c, d). H&E and Oil Red O staining both showed obvious liver pathological injury accompanied by the accumulation of fat and large distended lipid droplets in the liver of db/db mice compared with the C57 mice, which was remarkably reduced after polydatin treatment for 4 weeks (Fig. 3e).

**Table 1:** Polydatin attenuated serum lipid levels of db/db mice after 4 weeks of polydatin treatment (n = 6–7)

Group	Dose (mg/kg)	TC (mmol L^−1^)	TG (mmol L^−1^)	LDL-C (mmol L^−1^)	HDL-C (mmol L^−1^)
C57		2.18 ± 0.16	1.76 ± 0.26	0.49 ± 0.04	1.05 ± 0.08
db/db		4.06 ± 0.27^bbb^	2.87 ± 0.89	0.64 ± 0.08^bb^	1.97 ± 0.23^bbb^
Polydatin	100	3.24 ± 0.56^abb^	1.65 ± 0.61^a^	0.56 ± 0.06^a^	1.62 ± 0.31^abb^
Pioglitazone	10	3.89 ± 0.61	1.41 ± 0.23	0.70 ± 0.17	2.07 ± 0.32

Datas are represented as mean ± SD

Serum total cholesterol (TC), triglycerides (TG), low-density lipoprotein (LDL-C), and high-density lipoprotein (HDL-C) of db/db mice were measured after 4 weeks of polydatin treatment (n = 6–7)

^a^ *P* < *0.05* vs. C57

^bb^ *P* < *0.01*

^bbb^ *P* < *0.001* vs. db/db

**Fig. 4:**
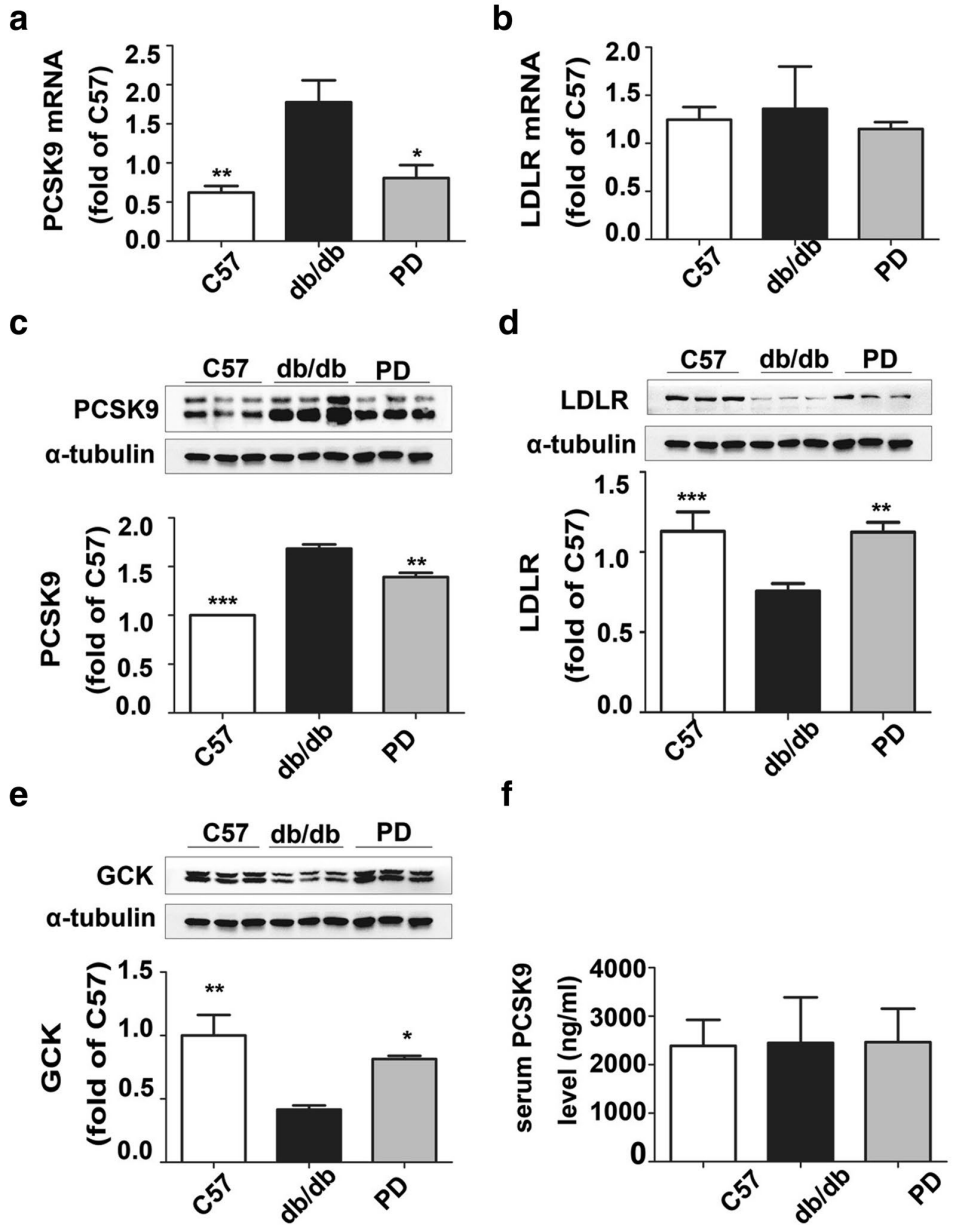
Polydatin inhibited PCSK9 level and upregulated the protein levels of LDLR and GCK. **a**, **b** PCSK9 mRNA and LDLR mRNA levels were detected by quantitative real-time PCR assay. **c**–**e** Protein levels of PCSK9, LDLR, and GCK were detected by Western blot analysis in mouse liver as described in “Methods”. Relative fold increases of each protein are shown (mean ± SD, n = 6). **f** ELISA assay was performed to detect the serum PCSK9 levels according to the manufacture’s instruction. *p < 0.05, **p < 0.01, and ***p < 0.001 vs. db/db

**Fig. 5:**
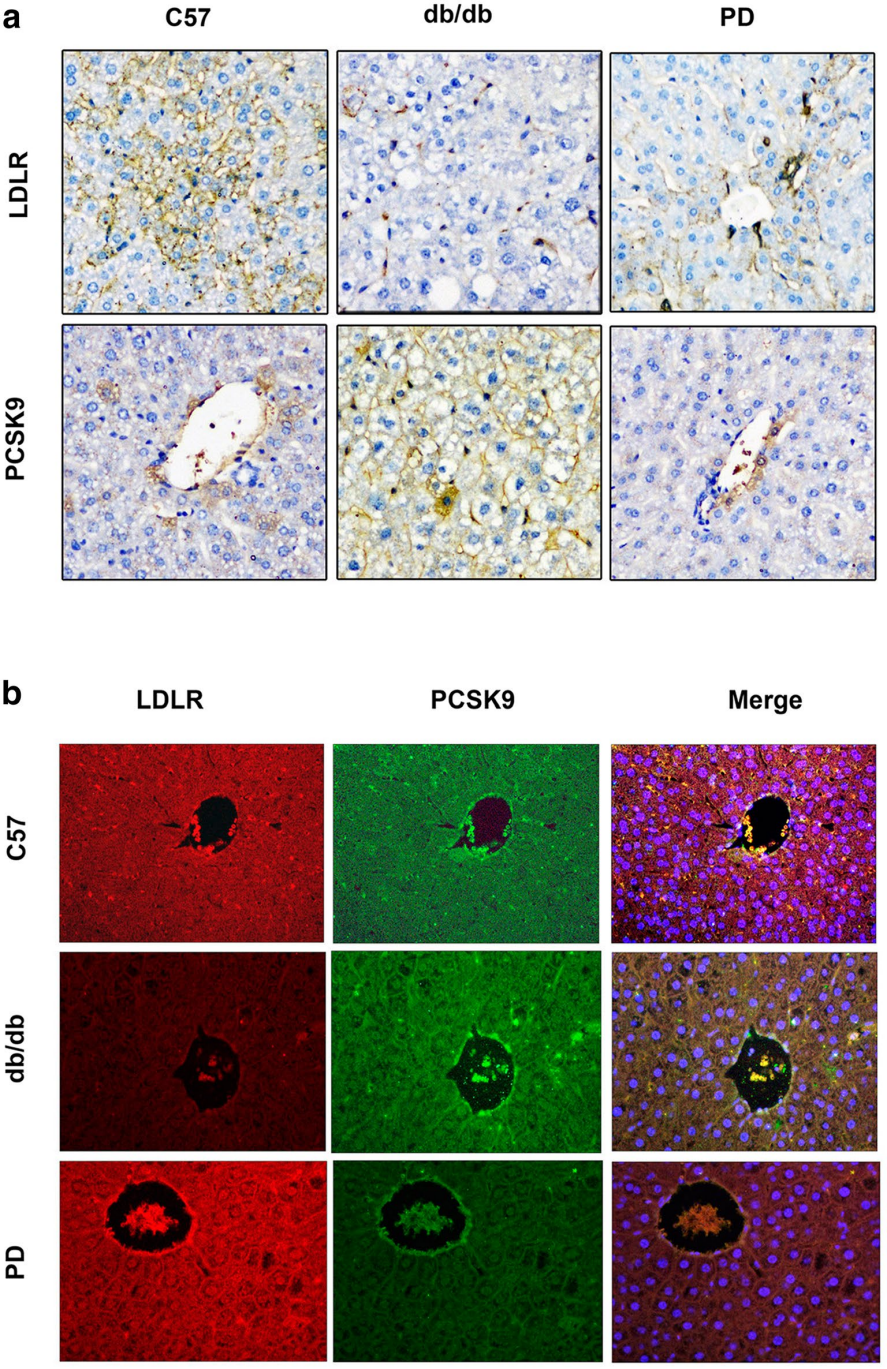
The relationship of LDLR and PCSK9 expression in the liver. **a** Immunohistochemistry results of LDLR and PCSK9 in the liver. For each representative section, the cells were considered to be positive for LDLR and PCSK9 if the cell bodies were stained brown and the relative contents were calculated by Image J analysis software. **b** Immunofluorescence staining revealed the relative expression and co-location of LDLR and PCSK9 in the liver. Six liver samples were used per group. (×400 magnification)

Polydatin significantly decreased PCSK9 mRNA levels (Fig. 4a) but had no effect on LDLR mRNA levels (Fig. 4b). Polydatin markedly inhibited PCSK9 expression (Fig. 4c) and increased protein levels of LDLR and GCK in db/db mice (Fig. 4d, e). ELISA assay was performed to measure the serum PCSK9 levels under polydatin-treatment conditions. As shown by Fig. 4f, there was no significant change in PCSK9 among the three groups. Immunohistochemistry staining showed that polydatin improved LDLR expression accompanied by the decrease in PCSK9 level (Fig. 5a) and immunofluorescence staining revealed the co-location of LDLR and PCSK9 in the liver to some extent (Fig. 5b), which was consistent with the western blot results.

Our vivo study better elucidated that polydatin could improve the lipid and glucose metabolism in db/db mice model possibly by inhibiting PCSK9.

### Molecular docking

**Fig. 6:**
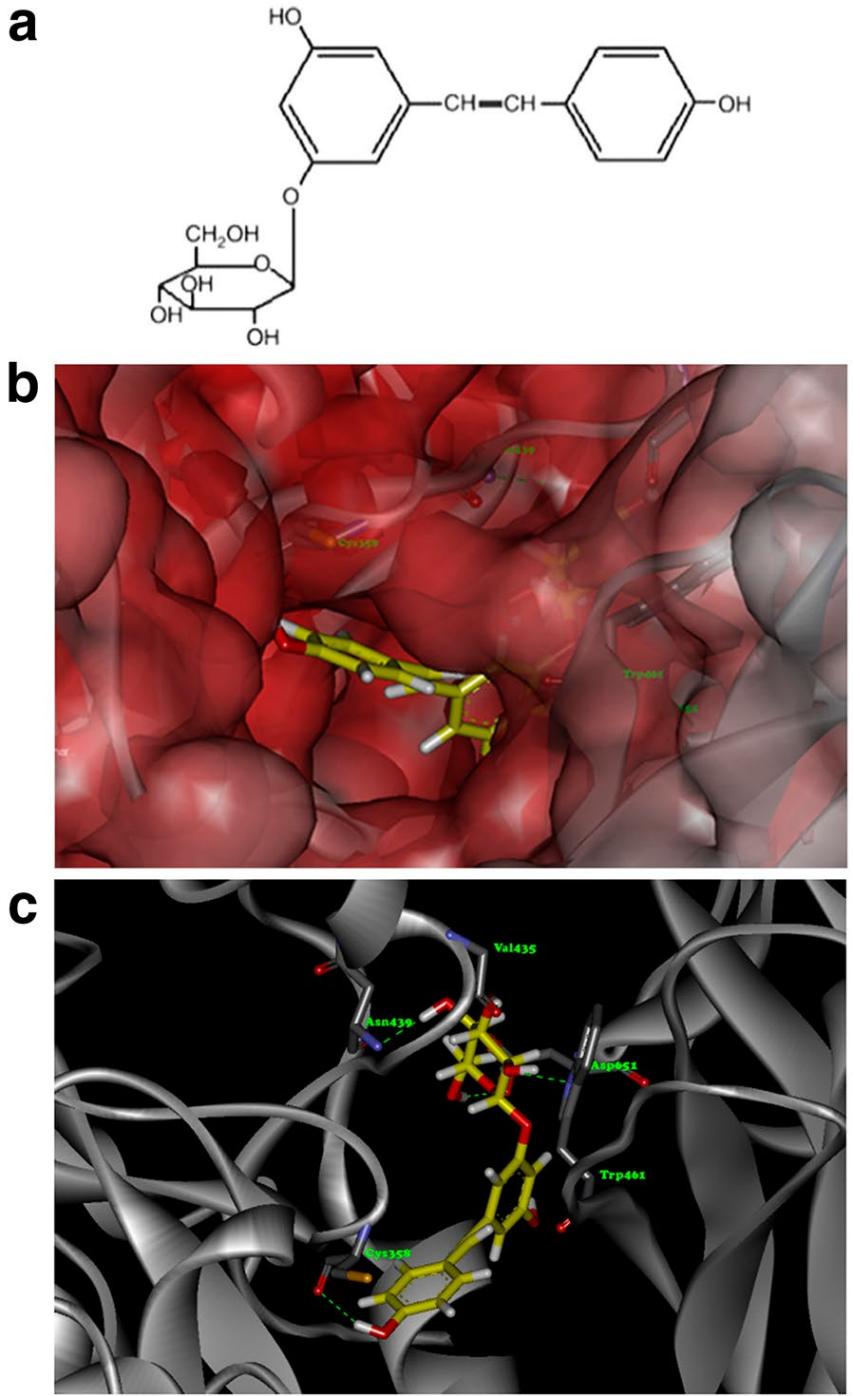
Molecular docking revealed a direct combination between polydatin and PCSK9 crystal structure (PDB code: 2p4e). **a** The chemical structure of polydatin, which contained several phenolic hydroxyl groups. **b** Docking pose of polydatin in the active pocket of the PCSK9 crystal structure. **c** Polydatin formed steady hydrogen bonds with PCSK9 in several amino residues consisting of Cys358, Val435, Asn439, and Asp651. Key residues are shown in stick representation. Carbon, oxygen, and hydrogen for the polydatin are colored *yellow*, *red*, and *silver*, respectively

To better figure out its interaction with PCSK9, polydatin was docked into the active pocket using Surflex-Dock in Sybyl 7.3.5. The molecular docking assay indicated that polydatin (Fig. 6a) could bind to the active pocket of the PCSK9 crystal structure (PDB code: 2p4e) (Fig. 6b) and formed steady hydrogen bonds with PCSK9 in several amino residues, including Cys358, Val 435, Asn439, and Asp651 (Fig. 6c). The interaction between PCSK9 and polydatin might inhibit the combination between PCSK9 and LDLR, and then decreased the degradation of LDLR.

## Discussion

The current study was undertaken to explore the effect and molecular mechanism of polydatin on lipid and glucose metabolism in type 2 diabetes. Polydatin, also known as piceid, is a main glucoside of resveratrol whose glucoside group bonded in the C-3 position substitutes with a hydroxyl group [20]. Both resveratrol and polydatin have the effects of anti-inflammation, anti-oxidation, cytoprotection in stress conditions, anti-hyperlipidemia, anti-hyperglycemia and many other cardiovascular protection merits [29, 32, 37–40], mainly through the signal pathways such as IKKs/NF-κB, Nrf2/ARE, Akt, AMPK, AMPK -Kir6.2/K-ATP [25, 32, 40–42]. The molecular mechanisms reported before are so widespread in almost all diseases that more immediate targets of polydatin should be explored to better explain the mode of drug action. According to our previous study, we found LDLR and GCK were significantly increased by polydatin in Streptozocin induced diabetic rats [32]. As reported, LDLR is the main receptor inducing cholesterol (especially LDL-C) clearance in circulation and consequently balances serum lipid level [43]. GCK can catalyze the phosphorylation of glucose to glucose 6-phosphate (G6P) as a key step of glycolysis, glycogen synthesis, and the pentose phosphate pathway [44]. And the expression of GCK is sharply decreased in diabetic mice [45]. Our data indeed indicated that polydatin can upregulate the expressions of LDLR and GCK both in vivo and vitro experiments, thereby improving the lipid and glucose metabolism.

While how polydatin regulates the expression of LDLR and GCK is unclear. Several studies have indicated that PCSK9 can bind to and induce the degradation of LDLR through both intracellular and extracellular pathways [46]. On the one hand, the PCSK9-LDLR complex is formed in the endoplasmic reticulum and then transfers directly from the Golgi network to the lysosome [47]. On the other hand, the secreted PCSK9 binds to LDLR on the cell surface, followed by internalization and degradation of LDLR in lysosomes [6, 13, 48, 49]. Therefore, PCSK9 arouses our interest and we make a reasonable speculation that polydatin might increase LDLR expression through decreasing PCSK9 levels. This will improve lipid metablism as well as insulin resistance, which will upregulate GCK levels and ameliorate hyperglycemia in a reciprocal way. Both vitro and vivo experiments are designed to demonstrate our inference.

In vitro, we firstly detected the levels of PCSK9, LDLR, and GCK using an insulin-resistant cell model induced by PA [32, 33]. As expected, we discovered an increase in PCSK9 expression in PA-induced insulin-resistant HepG2 cells that was reversed to control levels by polydatin, which was similar to the findings published in a previous study [50]. Further co-IP assay revealed decreases in LDLR (160 KD) content pulled down by PCSK9. These results indicate that polydatin may increase LDLR levels by repressing PCSK9 expression together with inhibiting the combination of PCSK9 and LDLR. SiRNA assay was performed to verify the above hypothesis. The upregulating effects of polydatin on LDLR and GCK were disappeared in the PA + PCSK9 knockdown group. Conversely, overexpression of PCSK9 wild-type plasmids caused a sharp decrease in LDLR and GCK levels compared with those in the empty vector group in insulin-resistant HepG2 cells. And restoration to control levels was achieved by polydatin. These results demonstrate that polydatin is involved in the regulation of LDLR and GCK by affecting PCSK9.

To better manifest our viewpoint, we further used a db/db mice model, which was mainly characterized by obesity, hyperglycemia, dyslipidemia, and insulin resistance [51, 52]. As expected, the polydatin treatment group showed significant hypoglycemia and lipid-lowering effects, consistent with a previous study [28]. PCSK9 mRNA and protein levels were enhanced in the liver tissues of db/db model. By comparison, LDLR did not show any change at the mRNA level, but exhibited a decrease in LDLR transcription in db/db mice, consistent with the InsR^−^/^−^ mice [18]. LDLR and GCK levels were both upregulated while the PCSK9 mRNA and protein levels were suppressed in db/db mice after polydatin treatment, corresponding to the vitro study. Immunohistochemistry and double immunofluorescence staining demonstrated the same results, and indicated the co-location of LDLR with PCSK9 on the cell surface to some extent, consistent with a previous study [47]. Many studies indicate that the function of PCSK9 as a secreted factor is physiologically significant [13, 16], thus we explored whether serum PCSK9 levels were affected by polydatin. We detected serum PCSK9 by ELISA and found no obvious change in the db/db mice group compared with that of the C57 group; such a result corresponds to previous reports [17, 53]. Different from statins’ upregulated effect of serum PCSK9 level [54], polydatin showed no significant influence on serum PCSK9 level. Our results imply that polydatin may enhance hepatic LDLR levels by decreasing PCSK9 contents and inhibit the PCSK9-LDLR complex formed directly in the liver rather than an extracellular route.

Both in vitro and in vivo studies revealed that polydatin ameliorates lipid and glucose metabolism disorders in a manner that is closely linked to PCSK9. However, the mechanism underlying the influence of polydatin on PCSK9 remains unknown. Hence, a molecular docking assay was performed, and several hydrogen bonds were observed to be formed between polydatin and the active pocket of the PCSK9 crystal structure. These results indicate that polydatin may bind to PCSK9 and change the conformation of PCSK9, thereby blocking the interaction between PCSK9 and LDLR and the degradation of LDLR. The results of this work provide a suitable theoretical basis for polydatin in lipid and glucose metabolism regulation by interfering with PCSK9.

## Conclusions

Polydatin ameliorates lipid and glucose metabolism via a mechanism that is closely relevant to its binding and blocking effect on PCSK9. Our study provides a new basis for further exploration of the therapeutic potentials of polydatin in the intervention and prevention of insulin resistance and regulation of lipid and glucose metabolism for future clinical use.

## Abbreviations

FBG: fasting blood glucose
GCK: glucokinase
IR: insulin resistance
LDLR: low-density lipoprotein receptor
PA: palmitic acid
PD: polydatin
Piog: pioglitazone
PCSK9: serine protease proprotein convertase subtilisin/kexin type 9
